# Supplementary material for: Myogenin controls via AKAP6 non-centrosomal microtubule-organizing center formation at the nuclear envelope
Source: eLife. 2021 Oct 4;10:e65672. doi: 10.7554/eLife.65672 (PMC8523159; doi:10.7554/eLife.65672)

Becker R *et al.*, Figure 2 - source data 2

Uncropped gel images for Figure 2J. The bands shown in panel 2J are marked by the dashed red line.

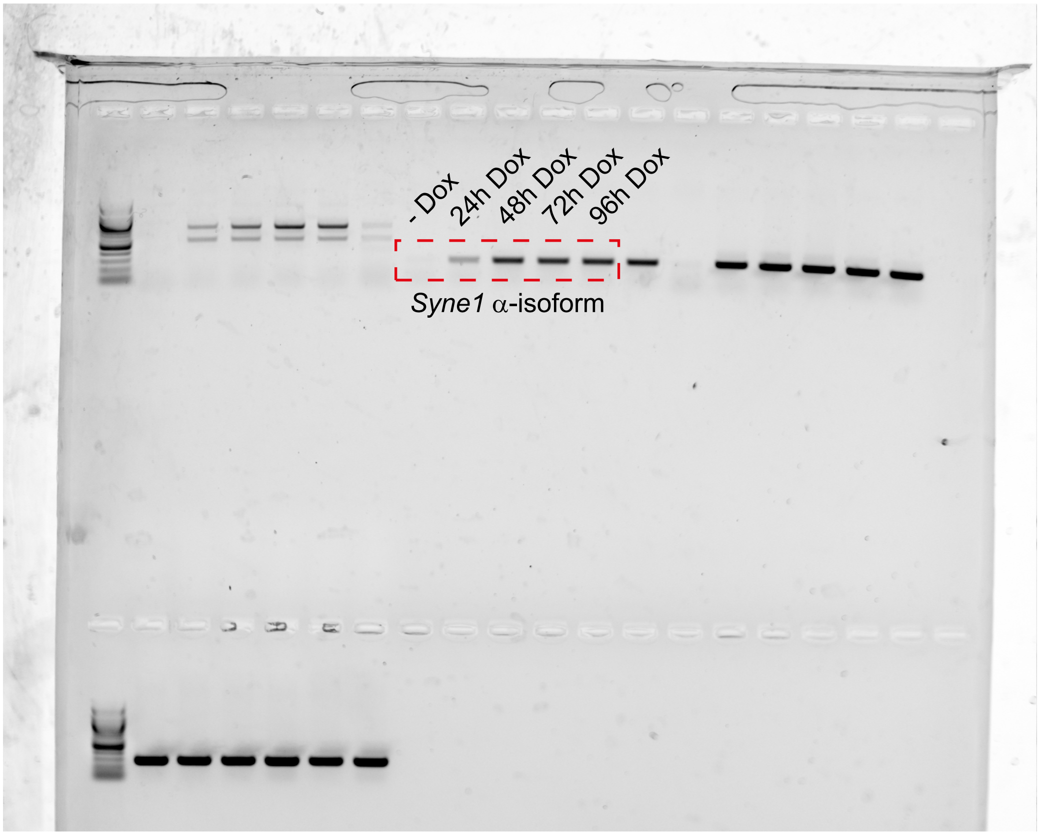

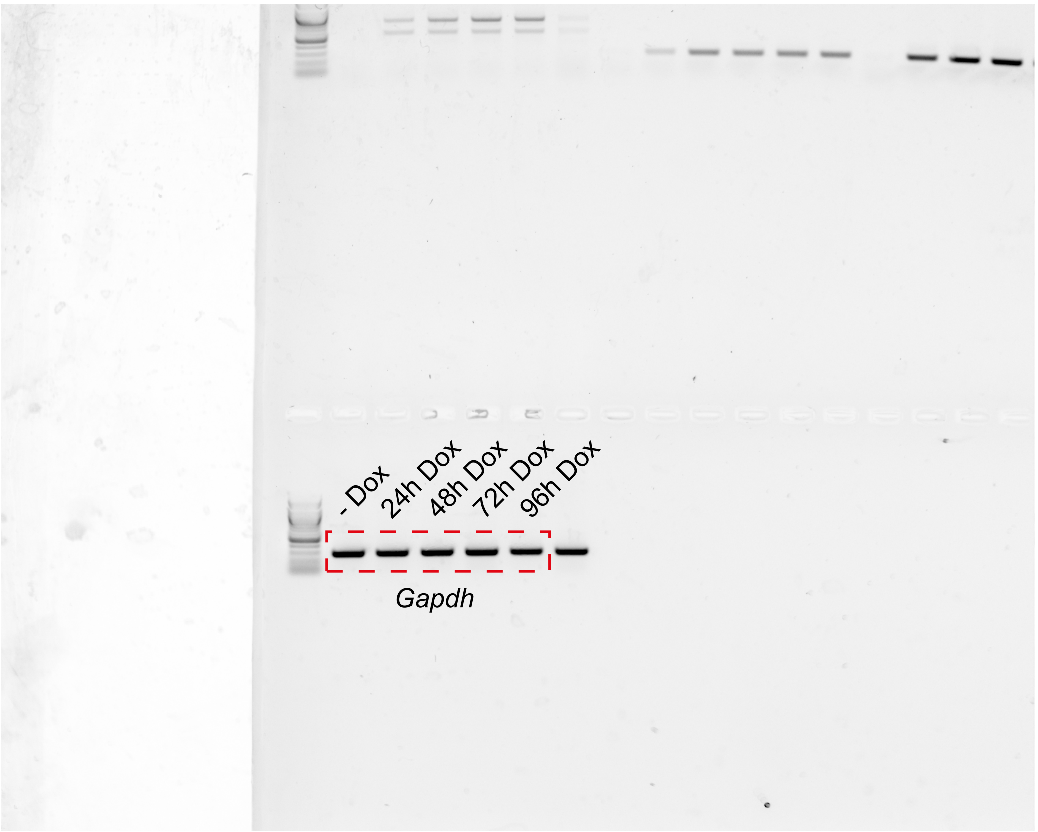

Supplement: Figure 2—source data 2. [file elife-65672-fig2-data2.zip › Figure 2 Source data 2/BeckerR_Figure 2 - source data 2.pdf]
