# Supplementary figures and images for: Myogenin controls via AKAP6 non-centrosomal microtubule-organizing center formation at the nuclear envelope
Source: eLife. 2021 Oct 4;10:e65672. doi: 10.7554/eLife.65672 (PMC8523159; doi:10.7554/eLife.65672)

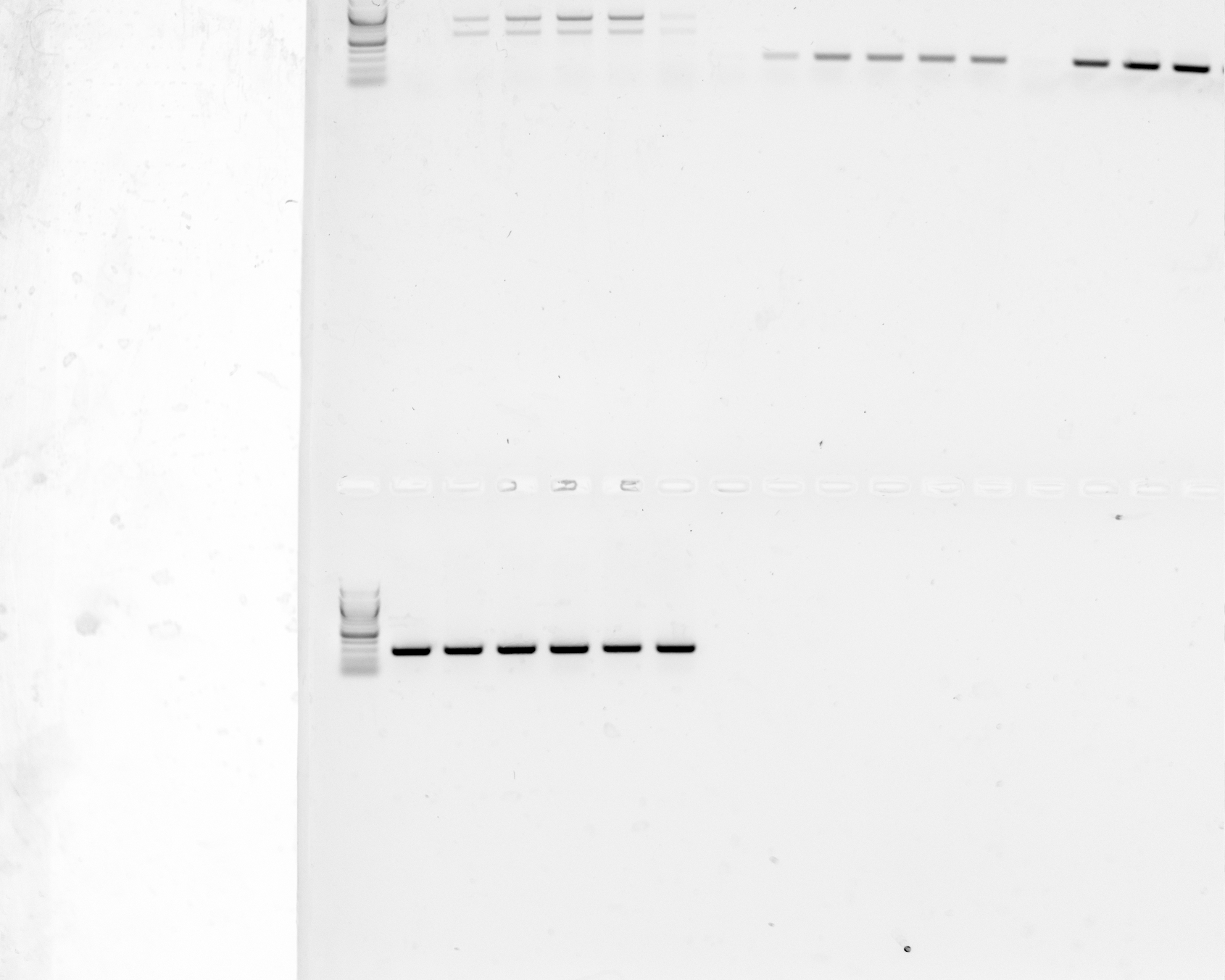

Supplement: Figure 2—source data 2. [file elife-65672-fig2-data2.zip › Figure 2 Source data 2/Gapdh raw.tif]

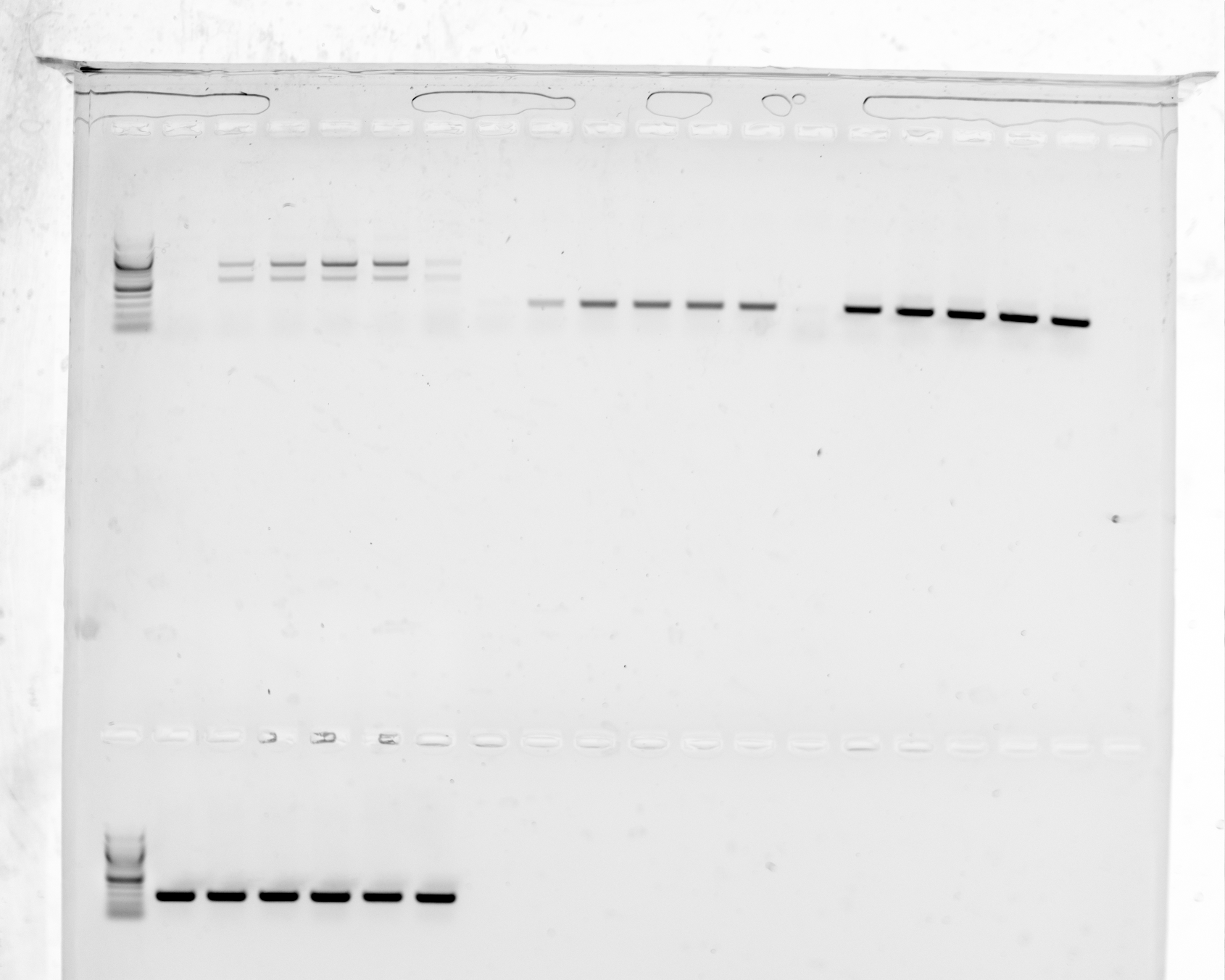

Supplement: Figure 2—source data 2. [file elife-65672-fig2-data2.zip › Figure 2 Source data 2/Syne1 alpha-isoform raw.tif]

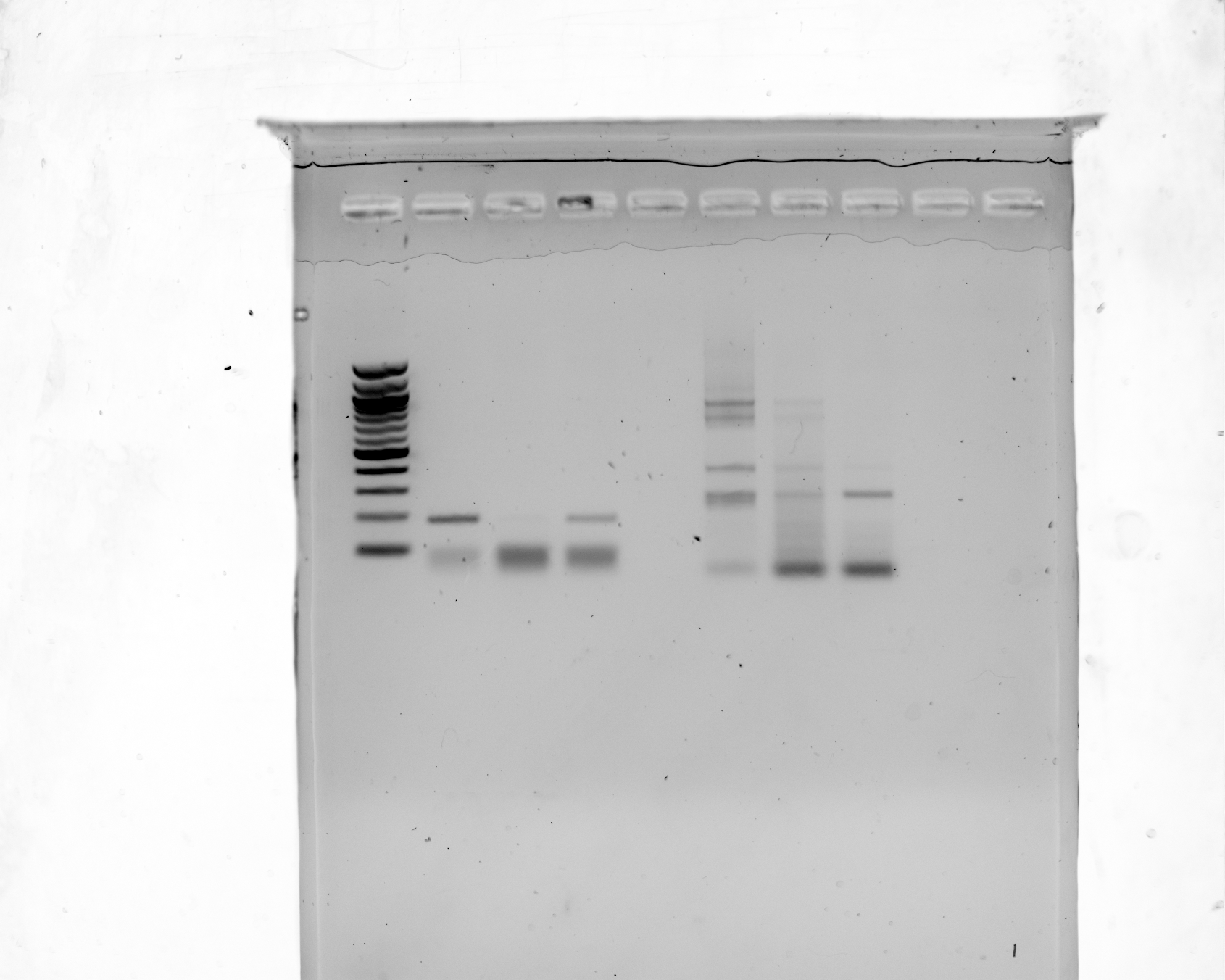

Supplement: Figure 2—source data 3. [file elife-65672-fig2-data3.zip › Figure 2 source data 3/alpha-isoform ChIP PCR raw.tif]

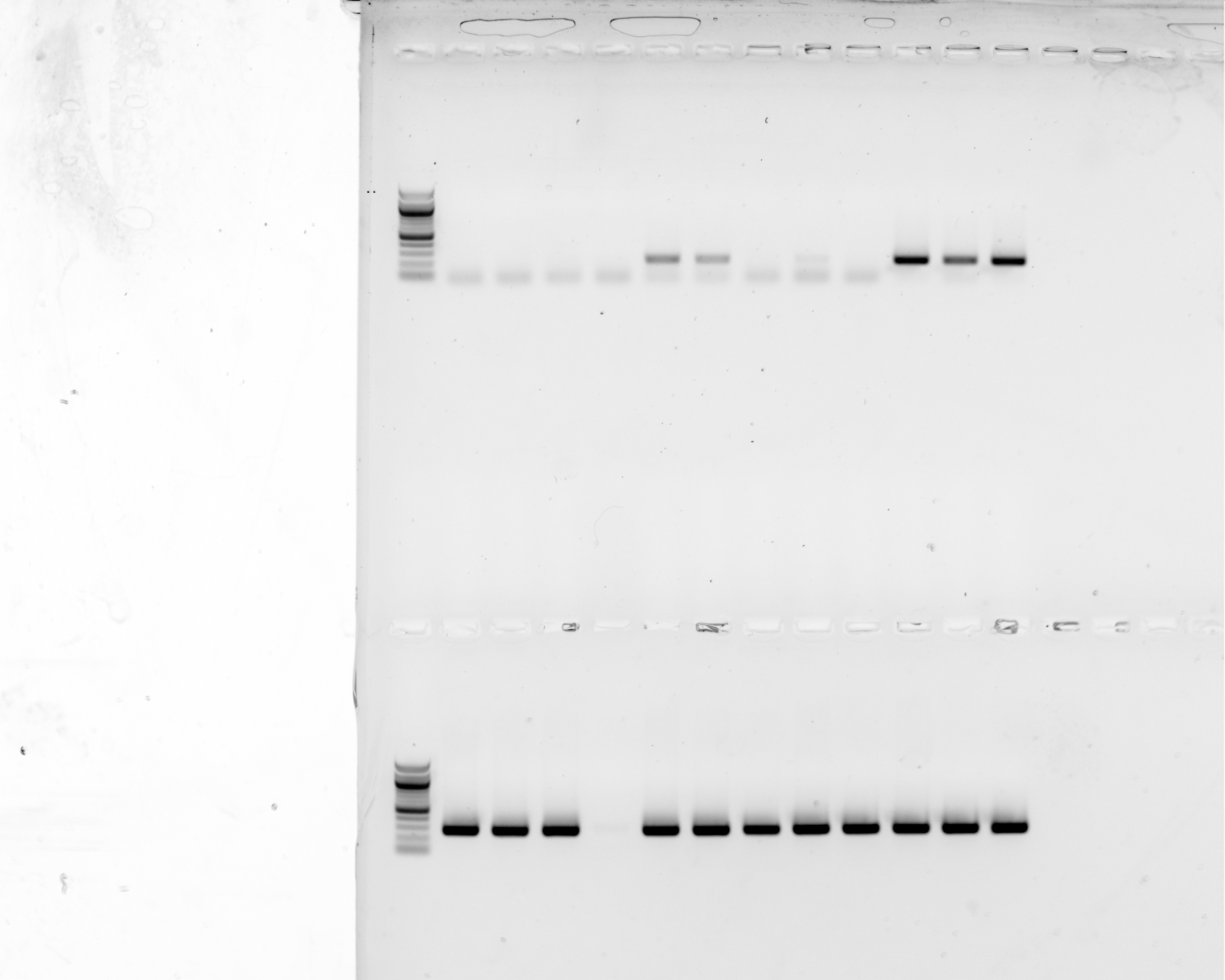

Supplement: Figure 2—figure supplement 1—source data 1. [file elife-65672-fig2-figsupp1-data1.zip › FPHAVW~B/Myod1 Gapdh raw.tif]

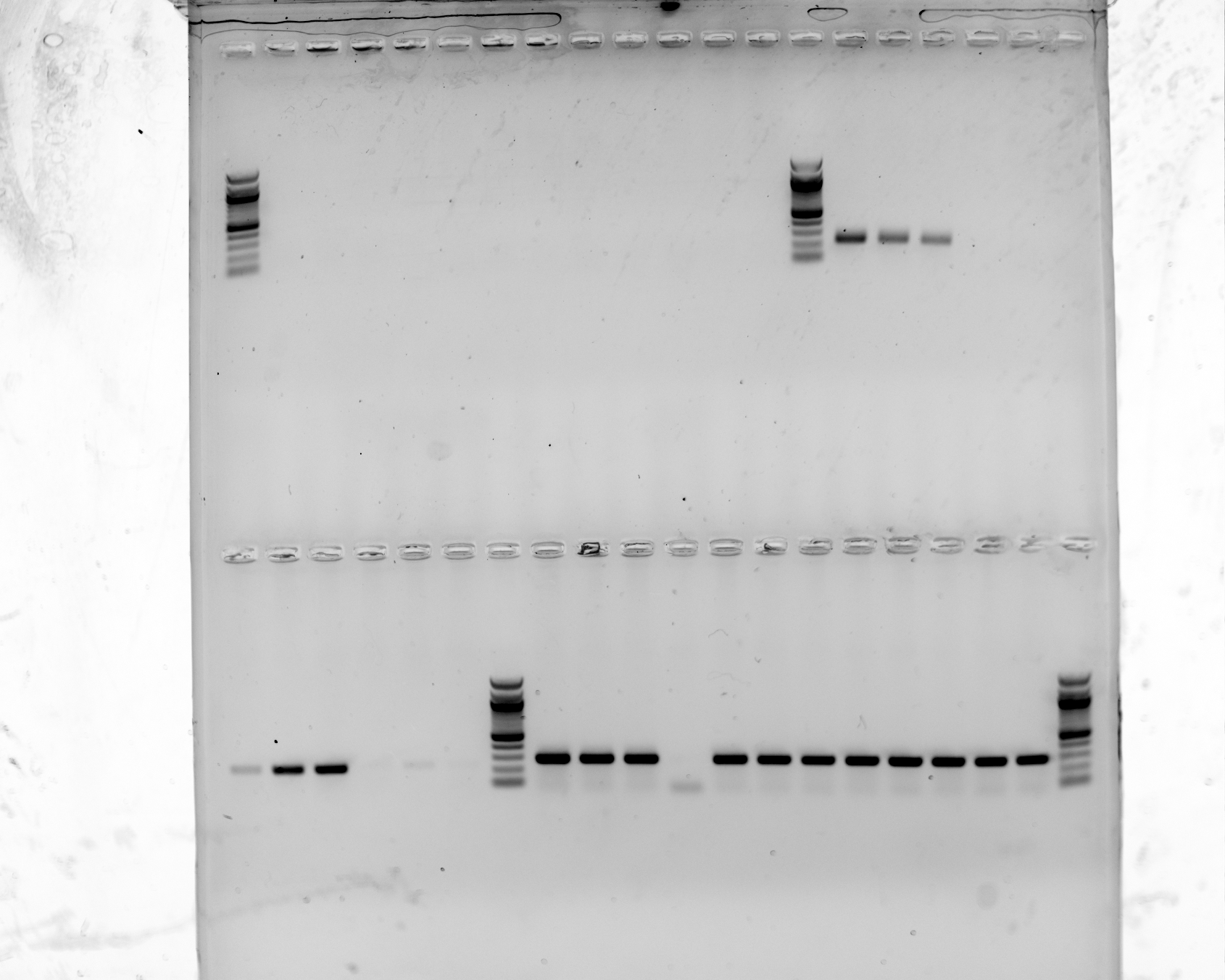

Supplement: Figure 2—figure supplement 1—source data 1. [file elife-65672-fig2-figsupp1-data1.zip › FPHAVW~B/Myog Gapdh raw.tif]

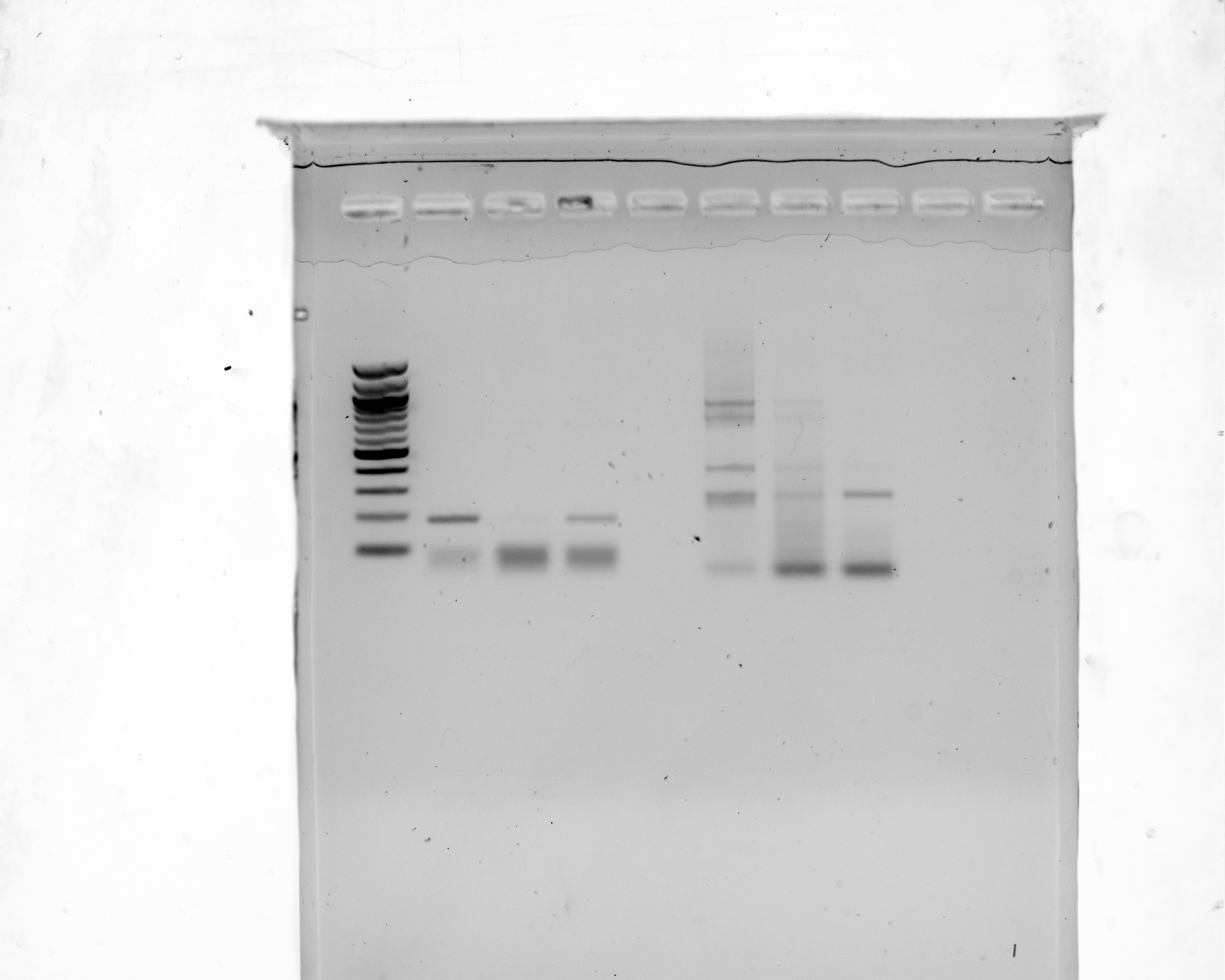

Supplement: Figure 4—source data 1. [file elife-65672-fig4-data1.zip › Figure 4 source data 1/Akap6 beta-isoform ChIP PCR raw.tif]

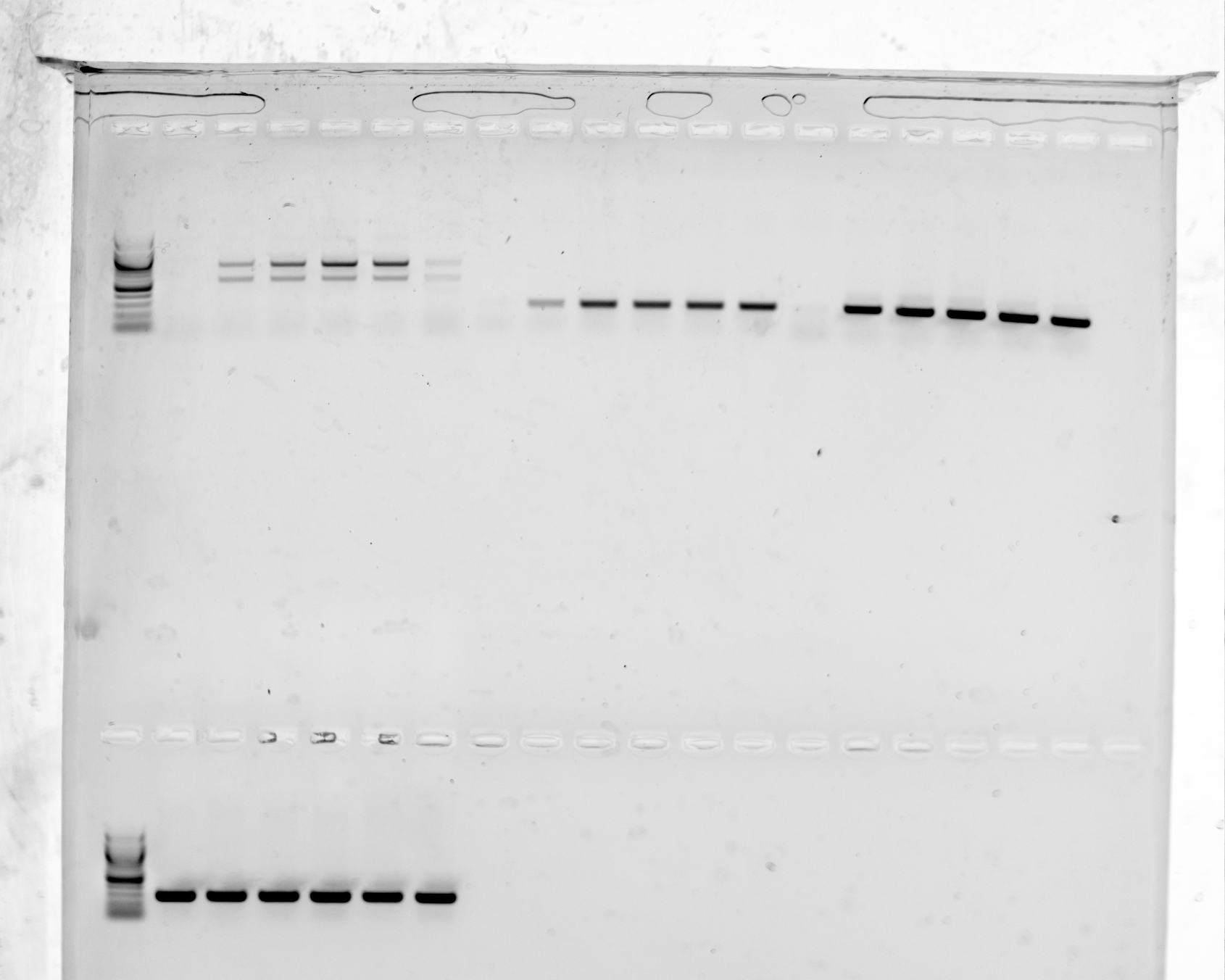

Supplement: Figure 4—source data 2. [file elife-65672-fig4-data2.zip › Figure 4 source data 2/Akap6 beta-isoform raw.tif]

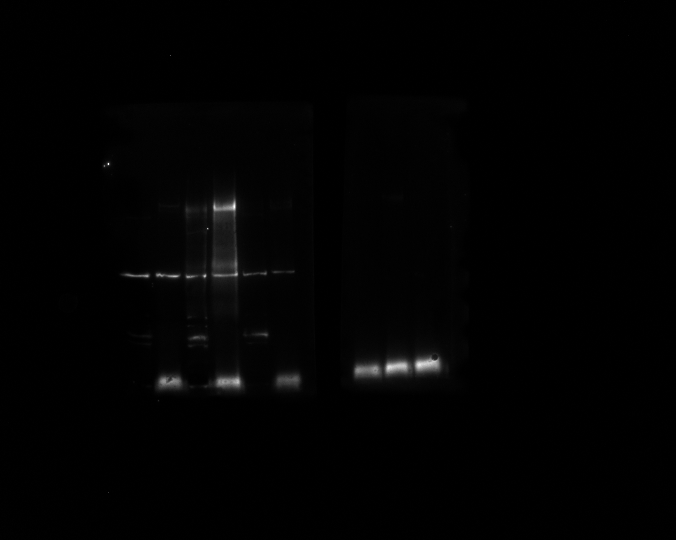

Supplement: Figure 5—source data 2. [file elife-65672-fig5-data2.zip › Figure 5 source data 2/left blot AKAP6_Chemiluminescence_raw.tif]

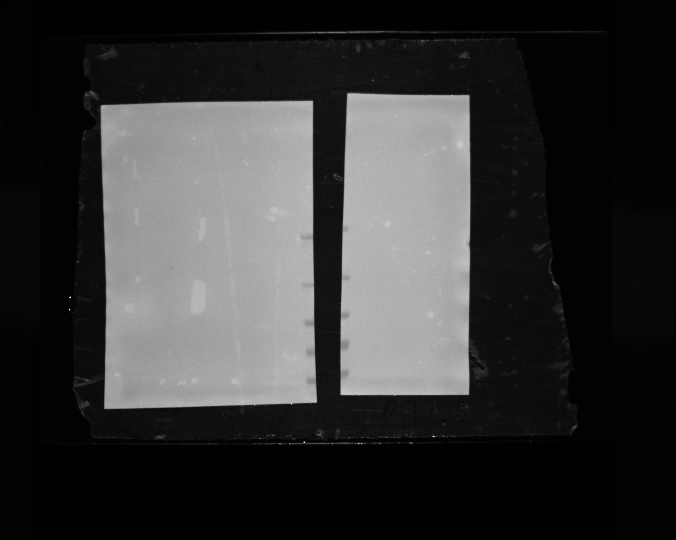

Supplement: Figure 5—source data 2. [file elife-65672-fig5-data2.zip › Figure 5 source data 2/left blot AKAP6_Membrane with marker_raw.tif]

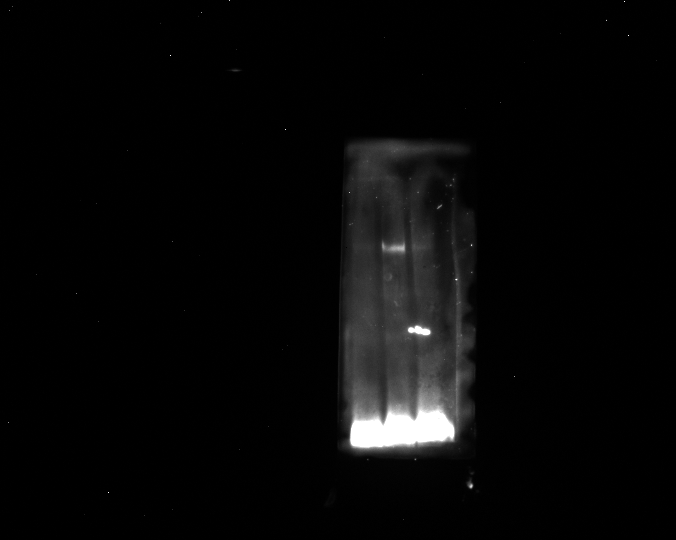

Supplement: Figure 5—source data 2. [file elife-65672-fig5-data2.zip › Figure 5 source data 2/right blot PCM1_Chemiluminescence_raw.tif]

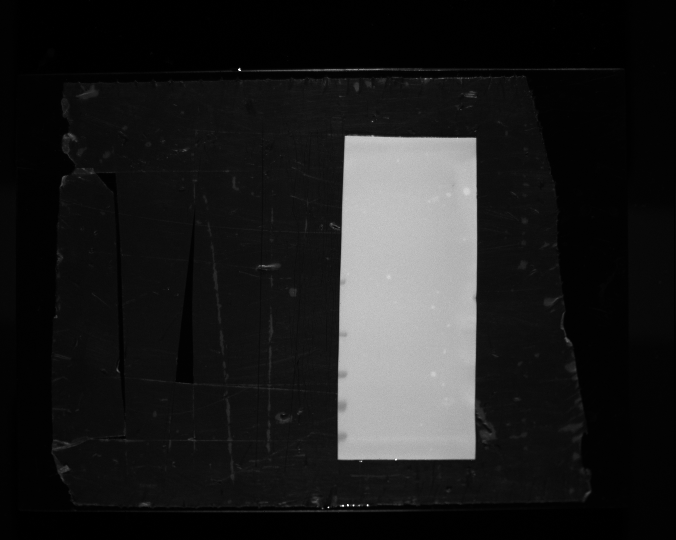

Supplement: Figure 5—source data 2. [file elife-65672-fig5-data2.zip › Figure 5 source data 2/right blot PCM1_Membrane with marker_raw.tif]
