# Supplementary material for: Myogenin controls via AKAP6 non-centrosomal microtubule-organizing center formation at the nuclear envelope
Source: eLife. 2021 Oct 4;10:e65672. doi: 10.7554/eLife.65672 (PMC8523159; doi:10.7554/eLife.65672)

Becker R *et al.*, Figure 2 - source data 3

Uncropped gel images for Figure 2K. The bands shown in panel 2K are marked by the dashed red line.

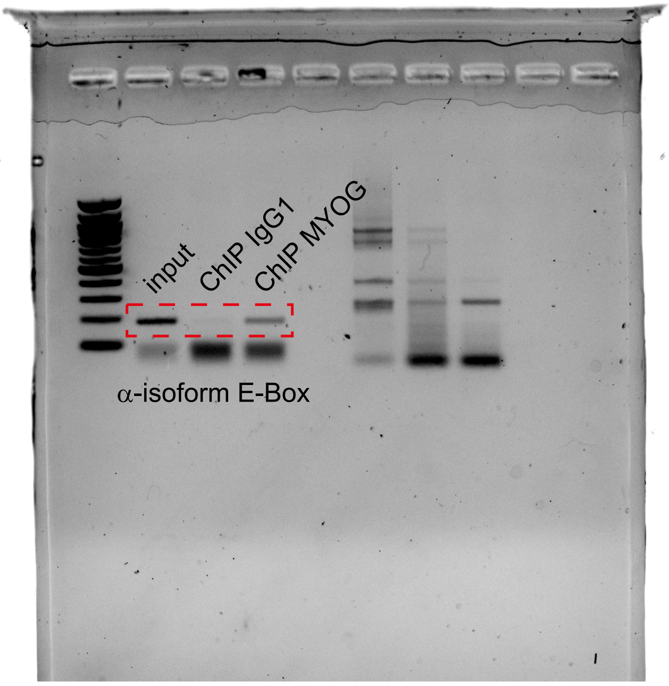

Supplement: Figure 2—source data 3. [file elife-65672-fig2-data3.zip › Figure 2 source data 3/BeckerR_Figure 2 - source data 3.pdf]
