# Supplementary material for: Myogenin controls via AKAP6 non-centrosomal microtubule-organizing center formation at the nuclear envelope
Source: eLife. 2021 Oct 4;10:e65672. doi: 10.7554/eLife.65672 (PMC8523159; doi:10.7554/eLife.65672)

# Becker R *et al.*, Figure 2 - figure supplement 1 source data 1

Uncropped gel images for Figure 2 - figure supplement 1C. The bands shown in panel 1C are marked by the dashed red line.

left gel images

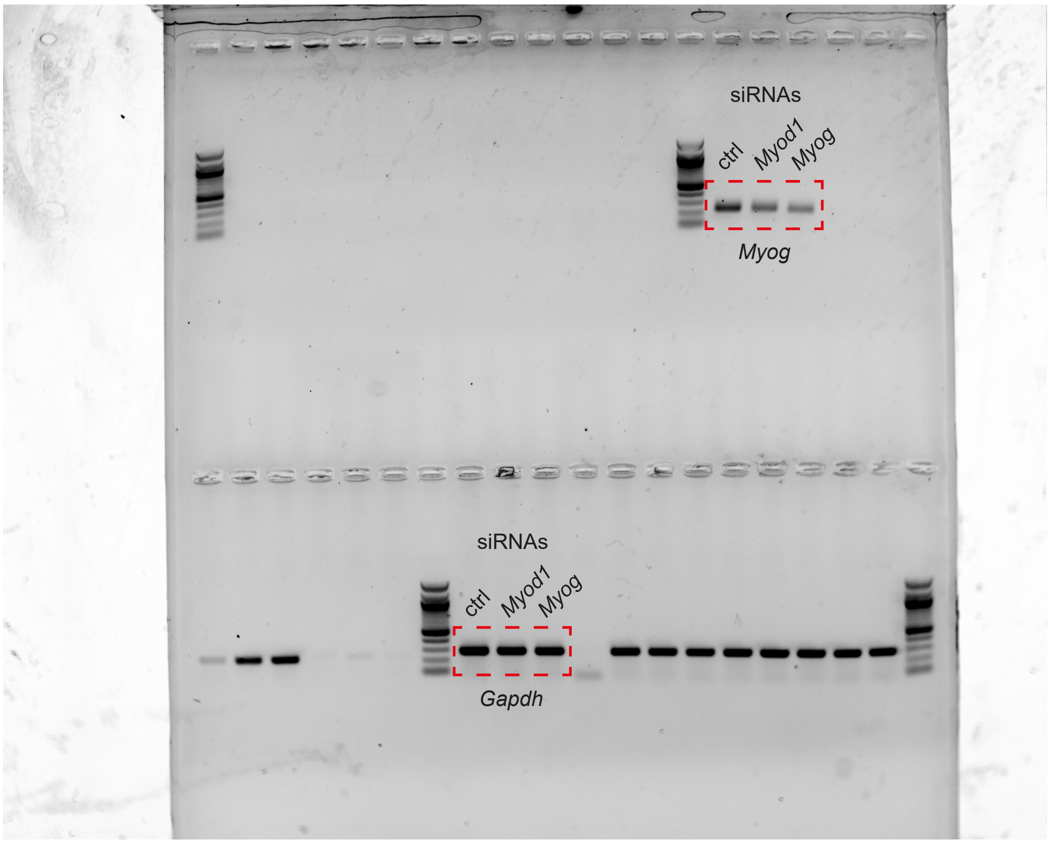


right gel images

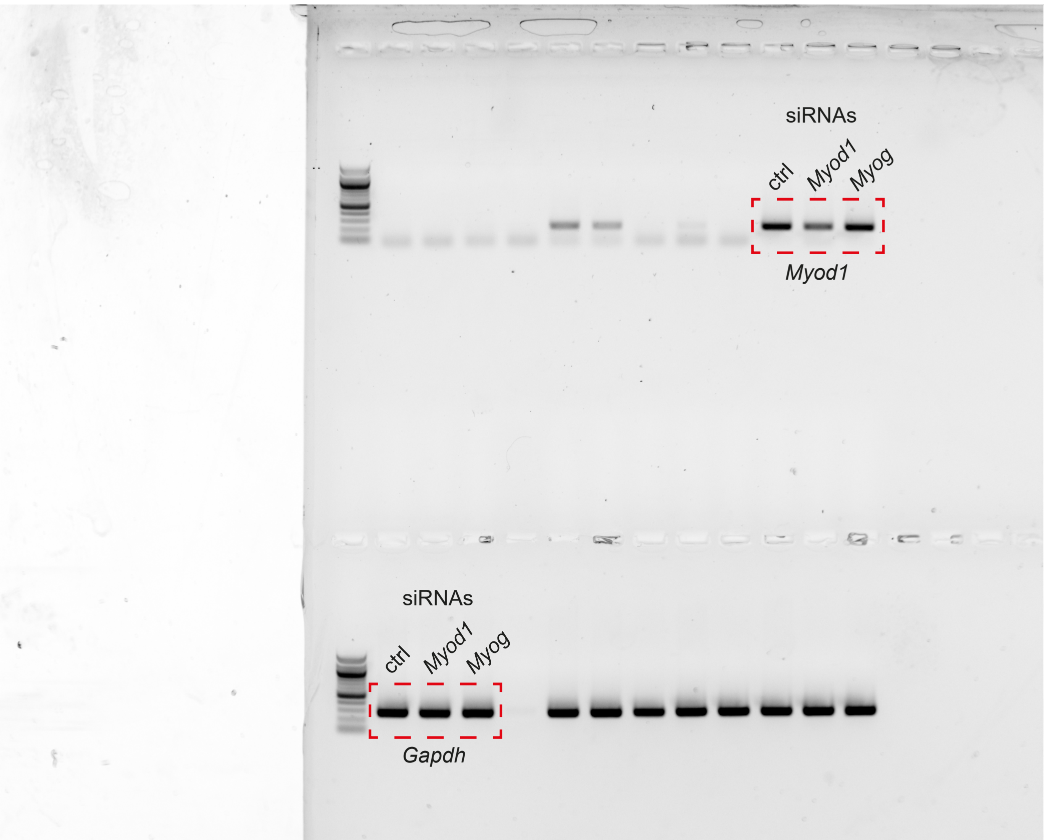

Supplement: Figure 2—figure supplement 1—source data 1. [file elife-65672-fig2-figsupp1-data1.zip › FPHAVW~B/BeckerR_Figure 2 - figure supplement 1 - source data 1.pdf]
