# Supplementary material for: Myogenin controls via AKAP6 non-centrosomal microtubule-organizing center formation at the nuclear envelope
Source: eLife. 2021 Oct 4;10:e65672. doi: 10.7554/eLife.65672 (PMC8523159; doi:10.7554/eLife.65672)

Becker R *et al.*, Figure 4 - source data 2

Uncropped gel images for Figure 4D. The bands shown in panel 4D are marked by the dashed red line.

upper panel

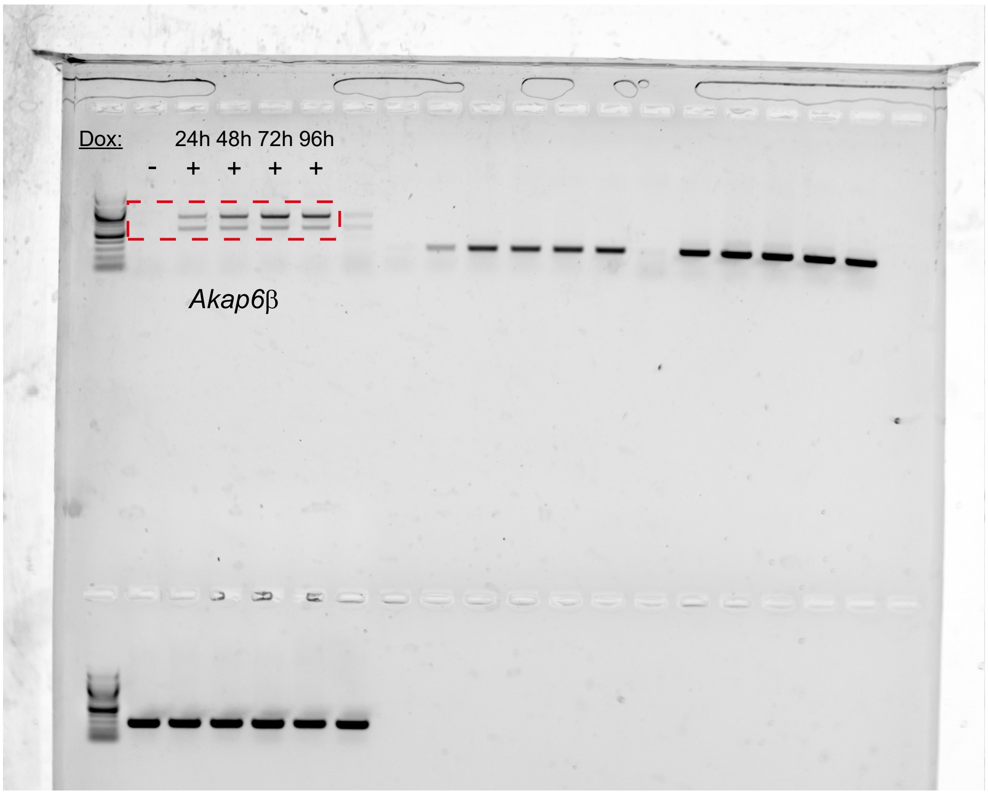

lower panel

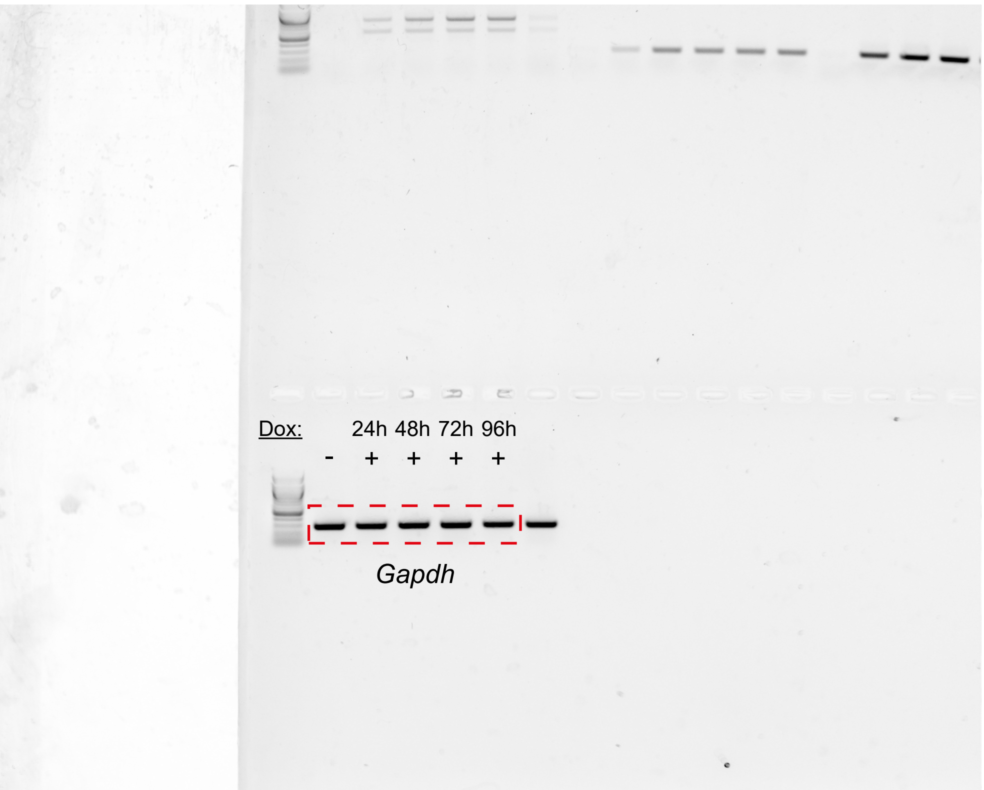

Supplement: Figure 4—source data 2. [file elife-65672-fig4-data2.zip › Figure 4 source data 2/BeckerR_Figure 4 - source data 2.pdf]
