# Supplementary material for: Myogenin controls via AKAP6 non-centrosomal microtubule-organizing center formation at the nuclear envelope
Source: eLife. 2021 Oct 4;10:e65672. doi: 10.7554/eLife.65672 (PMC8523159; doi:10.7554/eLife.65672)

Becker R *et al.*, Figure 5 - source data 2

Uncropped blots for panel 5G. Labels are the same as in the main figure. The top image shows the membrane and the dashed white line indicates where the membrane was cut along the molecular weight marker. The bands shown in panel 5G are marked by the dashed red line.

membrane

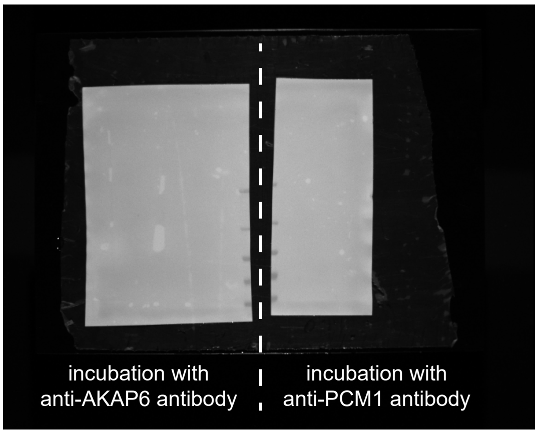

left blot

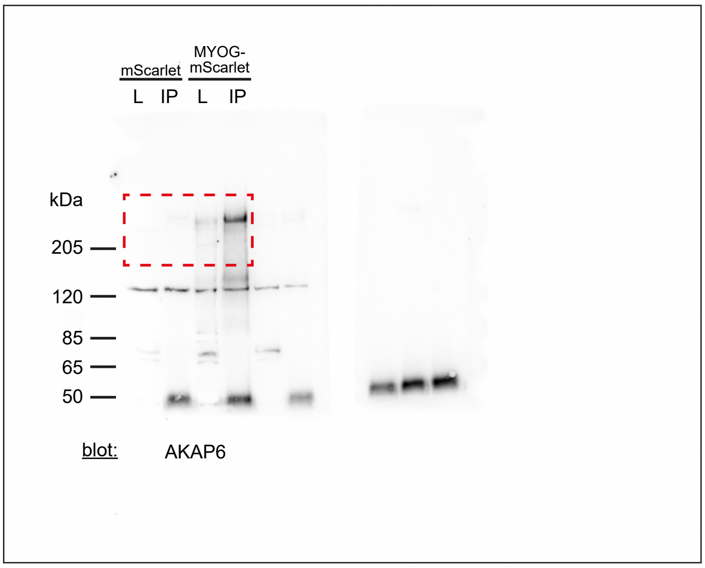

right blot

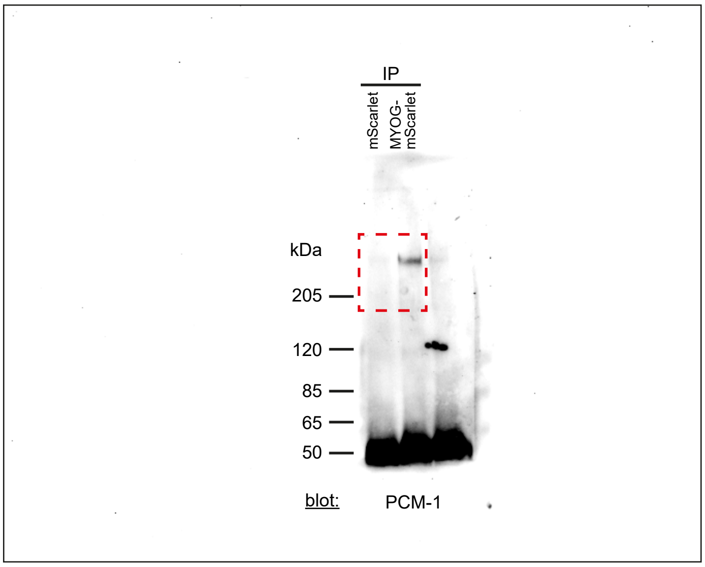

Supplement: Figure 5—source data 2. [file elife-65672-fig5-data2.zip › Figure 5 source data 2/BeckerR_Figure 5 - source data 2 5G.pdf]
