# Supplementary material for: Myogenin controls via AKAP6 non-centrosomal microtubule-organizing center formation at the nuclear envelope
Source: eLife. 2021 Oct 4;10:e65672. doi: 10.7554/eLife.65672 (PMC8523159; doi:10.7554/eLife.65672)
Supplement: Supplementary file 2. [file elife-65672-supp2.docx]

Supplementary File 2 – List of oligonucleotides used for PCR and construct generation

| Oligonucleotides | Source | Sequence |
| --- | --- | --- |
| cloning human myogenin into pLenti CMVtight Blast DEST forward | this paper | agccgccaccATGGAGCTGTATGAGACATC |
| cloning human myogenin into pLenti CMVtight Blast DEST reverse | this paper | ctgccctctccactgcccatGTTGGGCATGGTTTCATC |
| mScarlet cloning into pLenti CMVtight Blast DEST forward | this paper | tcctggcccaATGGTGAGCAAGGGCGAG |
| mScarlet cloning into pLenti CMVtight Blast DEST reverse | this paper | ggccgccactgtgctggataTCACTTGTACAGCTCGTCCATGC |
| psiCHECK™-2 assembly cloning forward | this paper | gctagccaccatggcttc |
| psiCHECK™-2 assembly cloning reverse | this paper | gtcgagccatgtgagcaaaag |
| AKAP6α promoter fragment assembly cloning forward | this paper | ttttgctcacatggctcgacgcaaaccatgattattatctaagtc |
| AKAP6α promoter fragment assembly cloning forward | this paper | tggaagccatggtggctagcaggagatgccttgtttgac |
| AKAP6β promoter fragment assembly cloning forward | this paper | ttttgctcacatggctcgactacagagaattaatgcttagaaac |
| AKAP6β promoter fragment assembly cloning reverse | this paper | tggaagccatggtggctagcgcttgcagtgaactgtaatg |
| Syne1-giant promoter fragment assembly cloning forward | this paper | ttttgctcacatggctcgacactctgtctctttcaacccaaacatcatgaacc |
| Syne1-giant promoter fragment assembly cloning reverse | this paper | tggaagccatggtggctagcggctgtgccctccctccc |
| Syne1-α promoter fragment assembly cloning forward | this paper | ttttgctcacatggctcgacgtgtcagattctgactcattg |
| Syne1-α promoter fragment assembly cloning reverse | this paper | tggaagccatggtggctagctggtcatgcttctaaataaaac |

| RT-PCR primers | Source | Sequence |
| --- | --- | --- |
| murine Akap6β forward | this paper | TCTAAAGCAGTTAGGCCCACAG |
| murine Akap6β reverse | this paper | CTTCGGATGAGCTCGGGAAAT |
| murine nesprin-1α forward | this paper | GAAGGACTGAGCCTTTCGCTC |
| murine nesprin-1α reverse | this paper | GGGCTTGGCCAACTCTGAC |
| murine Myod1 forward | this paper | GCTCTGATGGCATGATGGAT |
| murine Myod1 reverse | this paper | CGACTCTGGTGGTGCATCT |
| murine myogenin forward | this paper | CCAACCCAGGAGATCATTTG |
| murine myogenin reverse | this paper | CAGGACAGCCCCACTTAAAA |
| Gapdh forward | this paper | CAGAAGACTGTGGATGGCCC |
| Gapdh reverse | this paper | AGTGTAGCCCAGGATGCCCT |

| ChIP-primers (RT- and qPCR) | Source | Sequence |
| --- | --- | --- |
| Akap6 β E box forward | this paper | GTTTCCTTGTGCAGCAGAGC |
| Akap6 β E box reverse | this paper | TGGTCTGTTGGTGTGGTGTT |
| Akap6 α E box forward | (S. W. Lee et al., 2015) | CCAGATTTTAACCCCAGAAG |
| Akap6 α E box reverse | (S. W. Lee et al., 2015) | AGTGCACAGACTAATAATCG |
| Syne1 α E box forward | this paper | TGCTCTTGGTGGACAAATGGT |
| Syne1 α E box reverse | this paper | TCTTTTGAGCCGTAACCTGCT |
| Syne1 giant E box forward | this paper | CGCTAGCGAACGCCTCTTC |
| Syne1 giant E box reverse | this paper | GGAGGGACAGATCCTTTAGGC |
| Syne 1 intron forward | this paper | TACATTTGCCTCGTGGCTGT |
| Syne 1 intron reverse | this paper | TGTGCTTTAGCCTGGAGTGG |
| Akap6 intron forward | this paper | CCTGTCCTTTCTGCGTCGAT |
| Akap6 intron reverse | this paper | GTAACCCGCCAGAGAAAACG |
| Desmin promoter forward | this paper | GTCTTCTGTCCTCTTGGGGCTGTCCA |
| Desmin promoter reverse | this paper | TGGAGTGGATGTGAAGATGGGTGAC |
